# Fall Detection System Based on Point Cloud Enhancement Model for 24 GHz FMCW Radar

**DOI:** 10.3390/s24020648

**Published:** 2024-01-19

**Authors:** Tingxuan Liang, Ruizhi Liu, Lei Yang, Yue Lin, C.-J. Richard Shi, Hongtao Xu

**Affiliations:** 1 State Key Laboratory of Integrated Chips and Systems, Fudan University, Shanghai 201203, China; 18112020032@fudan.edu.cn (T.L.);; 2 ICLegend Micro, Shanghai 201203, China; 3 Department of Electrical and Computer Engineering, University of Washington, Seattle, WA 98195, USA; cjshi.fudan@gmail.com

**Keywords:** radar, fall detection, machine learning

## Abstract

Automatic fall detection plays a significant role in monitoring the health of senior citizens. In particular, millimeter-wave radar sensors are relevant for human pose recognition in an indoor environment due to their advantages of privacy protection, low hardware cost, and wide range of working conditions. However, low-quality point clouds from 4D radar diminish the reliability of fall detection. To improve the detection accuracy, conventional methods utilize more costly hardware. In this study, we propose a model that can provide high-quality three-dimensional point cloud images of the human body at a low cost. To improve the accuracy and effectiveness of fall detection, a system that extracts distribution features through small radar antenna arrays is developed. The proposed system achieved 99.1% and 98.9% accuracy on test datasets pertaining to new subjects and new environments, respectively.

## 1. Introduction

According to a report from the World Health Organization, approximately 28–35% of older adults fall each year, leading to serious injury or death [1]. Therefore, intelligently detecting falls in indoor conditions can reduce the risk of the elderly injuring themselves.

Various technologies have been adopted to detect falls. Existing fall detection methods require wearable sensors [2]. Accelerometers have been widely used in wearable methods, and a velocity threshold can be set to detect fall events [3,4]; however, these may be forgotten because of their inconvenience. Vision-based methods eliminate the need to wear something, but they are costly, sensitive to the lighting conditions, and invade privacy [5,6]. Recently, radar sensors have become more popular in fall detection system due to the advantages compared with other sensing technologies: (a) convenience over wearable technologies [7]; (b) high sensitivity to motion compared to depth sensors in complex living environments and weak lighting conditions; (c) privacy compliance over vision sensors [8]; and (d) low hardware cost compared with other sensors [9]. Typical radars for human fall detection are continuous wave (CW) radars and frequency-modulated continuous wave (FMCW) radars. A CW radar signal was converted into the time–frequency domain and extracted artificial features for detecting a falling person [10,11]. Doppler-time signatures recorded in the CW signals were used for the training of a machine learning algorithm [12]. However, CW radar can only provide velocity information. Due to the lack of information richness, actions with similar postures, such as sitting and squatting, may lead to inaccuracies. A better choice is to use an FMCW radar, which can simultaneously provide the range, Doppler, and angle information of the targets and also high sensitivity to motion [13].

Traditionally, researchers have explored serval methods based on FMCW radars, which range from 57–85 GHz [14]. The Doppler information could describe the velocity attribute of a motion; thus, the range-Doppler map has been widely used in FMCW radar-based fall detection methods proposed in the literature [15,16,17,18]. The Doppler-time map, including time information, was directly used as a feature to detect fall events [18]. Many studies on FMCW radar-based fall detection rely on the time–frequency characteristics of the FMCW radar return signal, including the frequency magnitude, frequency ratio, and the duration of the motion [19]. However, similar to CW radar-based methods, these methods cannot provide spatial information, and similar motions may lead to inaccuracies. Micro-Doppler and spatial information have been used to achieve high accuracy, proving that deep learning methods are superior to traditional artificial feature extraction methods [20]. An FMCW radio has been used to obtain the 3D position information of the human body and heatmaps in both horizontal and vertical directions [17]. However, there is still a problem in combining 3D location information: achieving high angular resolution using radars with large antenna arrays.

To utilize 3D spatial information, recent innovations in human activity detection have explored point clouds from radar [21,22,23], in which each point contains a 3D position in space. However, in contrast to LiDAR and camera sensors, there are two main challenges in these studies: (1) the point clouds generated by mmWave radar are usually sparse and of low resolution, and (2) the point clouds include many ghost points caused by the multipath effect. As a result, the classification accuracy and reliability may be negatively affected. To address these challenges, several methods have been designed for use in conjunction with high-resolution radars. 12Txs-16Rxs antenna arrays have been used to generate high-quality point clouds [22]. Hawkeye generated 2D depth images using radar intensity maps obtained from SAR scans [23]. However, although large antenna arrays and SAR technologies can improve the resolution, they are still very slow and may not be practical in many applications that require a short response time and low-cost hardware. In addition, sparsity-related methods and deep learning-based methods have been used for point clouds’ quality enhancement [24]. Some sparsity-related methods, such as K-mean [25] and density-based spatial clustering of applications with noise (DBSCAN) algorithm [26], have been used to cluster firstly to remove outliers in the point clouds. However, these technologies could not adequately remove a sufficient number of outlier points. In recent studies, a few deep learning-based methods have been developed based on PointNet [27]. After learning a mapping from the noisy input, they can automatically generate a set of clean points. Inspired by the PointNet, PCN combined a permutation invariant and non-convolutional feature extractor to complete a point cloud from a partial input, and then used a refinement block to denoise the prediction to produce the final point cloud [28]. GPDNet was applicable to denoise point clouds based on graph-convolutional layers [29]. However, most of these methods used LiDAR or other sensor applications and extracted pointwise features. Hence, they may not be efficient for radar-based point clouds because of their very low resolution.

In this study, we propose an FMCW radar-based fall detection method that investigates 3D point clouds while operating at 24 GHz. These systems have not been studied well owing to hardware limitations. First, we obtained raw point clouds from the radar. We then designed a new model to transform the raw points into high-quality point clouds that are closer to the ground truth. Next, we estimated the distribution parameters in the point clouds for classification.

**Figure 1:**
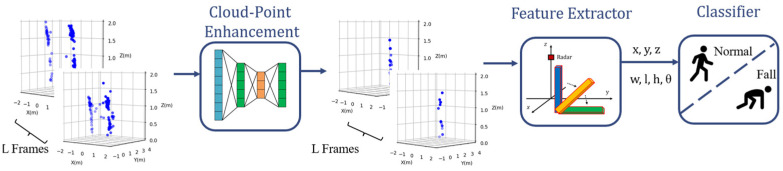
Proposed fall detection system based on PCE model.

The main contributions of this paper are as follows:

(1) We propose an efficient fall detection system that uses a small, low-cost radar. As shown in Figure 1, the novel framework is primarily composed of three parts: point cloud enhancement (PCE) for point cloud quality improvement, a feature extractor for human pose parameter extraction, and a classifier for classifying normal events and fall events;
(2) A PCE model is introduced to transform low-quality point clouds into high-quality point clouds and to reconstruct the shape of the point clouds using the shape of human body. A novel 3D point-box hybrid regression loss function based on pointwise and 3D bounding box features is proposed as a substitute for the traditional loss function;
(3) Our system works on sparse and noisy raw radar data without using expensive hardware or synthetic aperture radar (SAR) scans.

The remainder of this article is organized as follows. Section 2 provides an overview of the radar system and signal processing flow. In Section 3, the details of the proposed method are presented. The results are discussed in Section 4. Finally, Section 5 concludes this study.

## 2. Radar System Design

An FMCW radar is often used to estimate the range and velocity of a target using its radio frequency signals. Each transmitted signal $s\left(t\right)$ is a chirp signal, the analytical expression [30] of which is

(1) $$s\left(t\right)=\exp\left(j2\pi \left(f_{0}t+\frac{1}{2}Kt^{2}\right)\right)$$

where $f_{0}$ denotes the carrier frequency, and K is the chirp constant.

Referring to the Boulic model [31], the echo from the *i*th human body part is a time-delayed vision of the transmitted signal, and the received baseband signal can be expressed as

(2) $$S_{i}\left(t\right)=\exp\left(j2\pi f_{0}\left(t-\frac{2R_{i}}{c}\right)\right)$$

where $R_{i}$ is the distance between the $i^{th}$ ellipsoidal center and the radar, and c is the speed of light. The echo from the entire human body can be expressed as

(3) $$S_{all}\left(t\right)=\sum_{i=1}^{M}\eta_{i}S_{i}\left(t\right)$$

where $\eta_{i}$ is the attenuation coefficient, which is governed by the radar cross section for different body regions, and M is the number of scattering points of human body parts.

**Figure 2:**
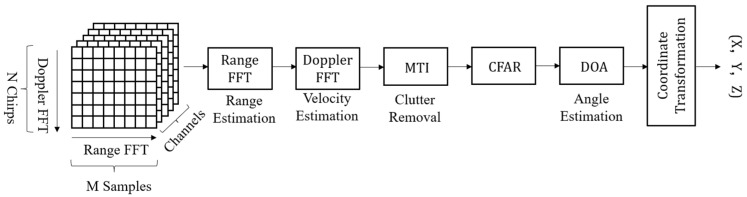
Signal processing flow.

As shown in Figure 2, the raw data from each channel generated a 3D data cube. FMCW radar signal processing started with the sampled echoes that were transferred to the range-Doppler matrix [32]. First, the range of the fast Fourier transform (FFT) assisted in estimating the range of the targets, and the second FFT determined the Doppler frequency. The moving target indicator distinguished the targets from the background. For more reliable detection, a constant false alarm rate was used to detect the targets against the background noise. A direction-of-arrival (DOA) estimation algorithm was used to estimate the angle of the target.

**Figure 3:**
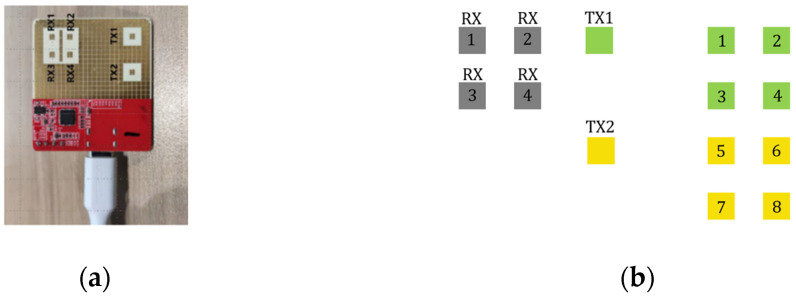
TDM-MIMO antenna arrays. (**a**) Radar diagram and real antenna array; (**b**) virtual antenna array.

For angle estimation, both 3D FFT and the multiple signal classification (MUSIC) algorithm [33] are popular methods. Compared with the MUSIC algorithm, 3D FFT has computing efficiency, but the resolution of 3D FFT is not sufficient for detecting closely spaced points. To obtain a better image of the human body and low computing cost, based on TDM-MIMO antenna arrays in Figure 3, after we obtained eight virtual receiver samples, a 3D FFT was used to obtain the azimuth angle $\theta_{az}$, and the MUSIC algorithm was used to estimate the elevation angle $\theta_{el}$. The output spherical coordinates were converted into Cartesian coordinates using transfer matrix T:

(4) $$\left[\begin{array}{c} x\\ y\\ z \end{array}\right]=\left[\begin{array}{ccc} 1 & 0 & 0\\ 0 & {cos\theta }_{t} & {sin\theta }_{t}\\ 0 & {-sin\theta }_{t} & {cos\theta }_{t} \end{array}\right]\left[\begin{array}{c} R{cos\theta }_{el}{sin\theta }_{az}\\ R{cos\theta }_{el}{cos\theta }_{az}\\ R{sin\theta }_{el} \end{array}\right]+\left[\begin{array}{c} 0\\ 0\\ h \end{array}\right]$$

where R is the distance between the target and the radar. After the transformation, we obtained the position values [x,y,z] of each point in each frame in Cartesian coordinates.

## 3. Proposed Method

**Figure 4:**
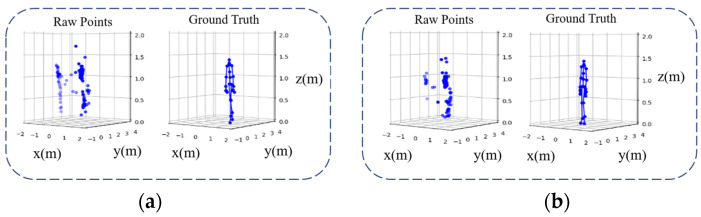
Raw point clouds in different environments. (**a**) Raw point clouds from radar collected in a relatively empty room; the number of points is 85. (**b**) Raw point clouds collected in a real bedroom; the number of points is 125.

The point clouds detected from the range-Doppler map are sensitive to the environment. Figure 4 shows different point distributions generated in different indoor environments, in which obstacles cause severe multipath interference and affect the performance of fall detection systems in new environments. 

**Figure 5:**
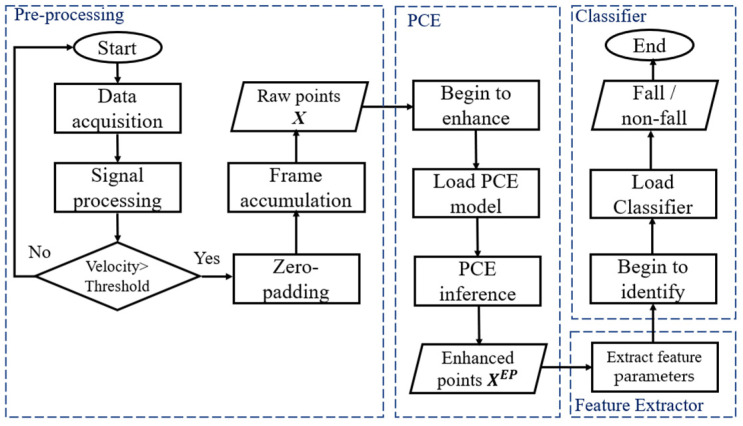
The flowchart of proposed system.

Therefore, we propose a fall detection method based on a PCE model. A flowchart of the proposed method is presented in Figure 5. The flow of proposed method consists of four steps: (1) to satisfy the input of the PCE model, the number of point clouds of a motion pattern could be extended to fixed number; (2) after raw point clouds from the radar in pre-processing, PCE model removes noise points and generates high-quality point clouds; (3) these point clouds are then fed into the feature extractor for human pose parameter extraction; (4) a lightweight classifier is used for classifying normal and fall events. In this section, the functionality of each component is described. More detailed descriptions are as follows.

### 3.1. Pre-Processing

The motion window accumulates *L* frames of the point cloud from the radar, where *L* is determined by the length of an action such as walking, squatting, and falling. For each action pattern, we extracted the 3D vectors of $\left[x,y,z\right]$ for each point in every frame. In each frame, the number of radar point clouds was random owing to the nature of the FMCW radar measurement. To satisfy the input of the PCE model, zero padding was used for data oversampling. Thus, the number of point clouds can be extended to a fixed number. We obtained the motion pattern

(5) $$X={\left\{{\left\{x_{m}^{i}\right\}}_{m=1}^{M}\right\}}_{l=1}^{L}$$

where *l* is the frame index of the motion, *M* is the number of points from radar in each frame, and $p_{m}^{l}$ is the $m^{th}$ point in the $l^{th}$ frame, which is a vector of $\left[x_{m}^{l},\;y_{m}^{l},\;z_{m}^{l}\right].$

Simultaneously, the Azure Kinect was used as a reference sensor because of its high precision in extracting human skeletal keypoints. Ignoring some inessential points, the desired 27 skeletal keypoints returned from Azure Kinect were also accompanied by a radar time stamp in system time and served to label the ground truth in the training process.

### 3.2. PCE Model

**Figure 6:**
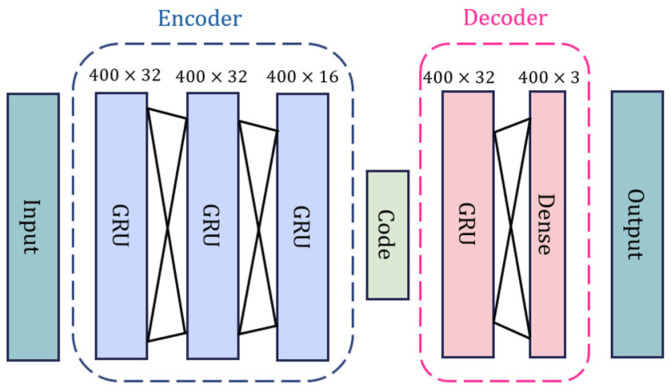
Proposed PCE model architecture.

Although point clouds have already been obtained from range-Doppler maps, the resulting points are still low-resolution, very noisy, and affect the classification accuracy and reliability. In addition, it is unnecessary for a fall detection system to increase the computational cost of reconstructing each point. Thus, to improve the quality of these point clouds and increase classification reliability, we propose a PCE model that aims to minimize the difference between the shapes of the radar point clouds and the ground truth. An overview of the proposed PCE architecture is shown in Figure 6.

#### 3.2.1. Encoder

The encoder is responsible for extracting global features from incomplete point clouds. As shown in Figure 6, the encoder comprises three consecutive gate recurrent units (GRU) [34] followed by a dense layer. Using GRU mechanisms in a machine learning network is a relatively new time-series architecture compared with recurrent neural networks (RNNs) and long short-term memories (LSTMs). GRUs can retain long-term dependencies and are also faster for training compared with traditional LSTMs because there are fewer gates to update. As shown in Figure 6, the units of the three GRU layers are 32, 32, and 16. The input data $X^{RP}=\left\{x_{1}^{RP},x_{2}^{RP},\ldots x_{N}^{RP}\right\}$ are first reconstructed by the hidden parts, and the output of the encoder is a series of vectors $\left\{h_{1},h_{2},\ldots h_{b}\right\}$. Thus, the encoder process is given by

(6) $$h_{b}=GRU\left(W_{e,b}X^{RP}+b_{e,b}\right)$$

where $W_{e,b}$ and $b_{e,b}$ are the encoder weight matrix and bias vector for the *bth* node (*b* = 1, 2, …*B*).

#### 3.2.2. Decoder

The decoder consists of three consecutive GRUs followed by a dense layer. The units of the three GRU layers are 32. The output from the GRU is

(7) $$u_{i}=GRU\left(W_{d,b}w_{i}+b_{d,b}\right)$$

where $W_{d,b}$ and $b_{d,b}$ are the decoder weight matrix and bias vector for the *bth* node (*b* = 1, 2…*B*), and ***wi*** is the input layer of the decoder. The recovered point clouds $\hat{X}$ through the dense layer is obtained via

(8) $$\hat{X}=W_{d}u_{i}+b_{d}$$

where $W_{d}$ and $b_{d}$ are the weights and biases of the output layer, respectively. After the sampling layer, an enhanced point cloud $X^{EP}$ is obtained.

#### 3.2.3. Loss Function

**Figure 7:**
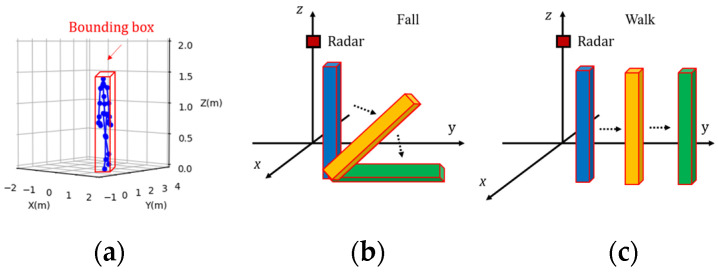
(**a**) Pose based on the 3D bounding box of a standing human body; (**b**) change in the 3D bounding box of a falling human body; (**c**) change in the 3D bounding box of a walking human body.

A simple method for extracting pointwise features in 3D space is supervised by the mean squared error (MSE). However, directly supervising the pointwise features of point clouds may not utilize 3D receptive field information. The observations in Figure 7 indicate that the change in the 3D receptive field bounding box of a falling human body is different from that of a walking human body. Specifically, changes in the bounding box of a human body are related to its pose. Therefore, we not only consider pointwise features but also detect fall events by characterizing the uniqueness of bounding box changes for such poses.

To use the bounding box of the radar point clouds for fall detection, the position and shape of the predicted box should be closely related to the corresponding ground truth. In this manner, we propose a 3D point-box hybrid regression loss to reduce the error between the predicted radar bounding box and ground truth. Previous studies [35,36] found intersection over union (IoU) loss and generalized IoU functions for 2D boxes using only the width and height of the boxes without considering the direction of the boxes. In addition, it is difficult to provide a specific formula describing the intersection between two 3D bounding boxes because a variety of cases must be considered. Some previous studies projected a 3D bounding box onto two 2D bounding boxes, but this did not increase the accuracy because of lack of direction [37].

**Figure 8:**
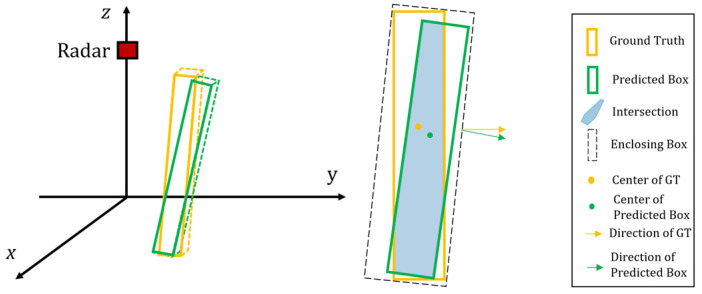
Example of a polygon formed by the overlap of the predicted box and ground truth. The 3D IoU can be approximately achieved by multiplying the length along the x-axis with the directed 2D IoU.

**Figure 9:**
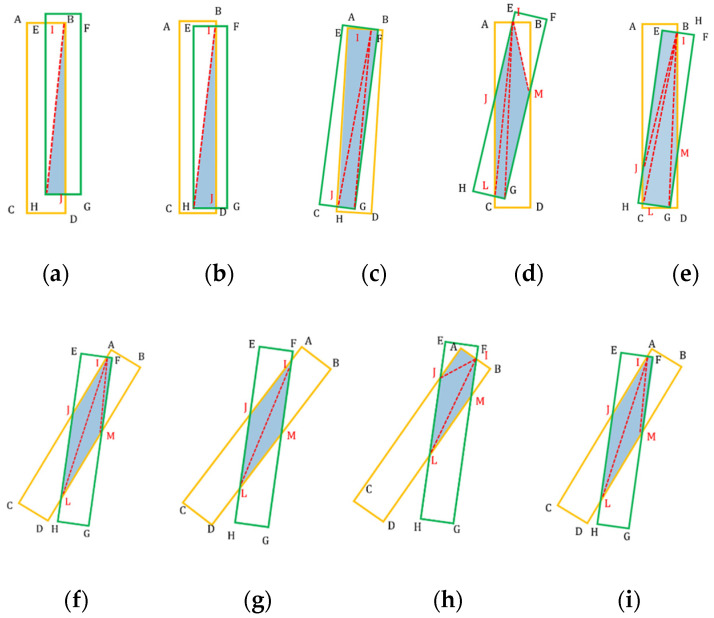
(**a**–**i**) are examples of the directed 2D IoU with different numbers of intersection points. The insertion area is obtained by summing the triangular areas.

In this study, because most of the measured human motion states were symmetrical along the x-axis (as shown in Figure 8), a 3D bounding box was obtained based on the 2D rotated IoU by multiplying the length along the x-axis. As shown in Figure 9, the intersection of the predicted box and ground truth included a variety of polygons, which was the sum of the areas of all the triangles. Therefore, the 3D IoU can be expressed as

(9) $${IoU}_{3D}=\frac{{Area}_{OL}\cdot x_{OL}}{{Area}_{r}\cdot x_{r}+{Area}_{GT}\cdot x_{GT}-{Area}_{OL}\cdot x_{OL}}$$

where ${Area}_{OL}$ is the area of overlap between the two boxes; ${Area}_{r}$ and ${Area}_{GT}$ are the areas of the predicted box from radar and ground truth, respectively; $x_{OL}$ is the overlap on the x-axis; and $x_{r}$ and $x_{GT}$ are the lengths of the predicted box and ground truth along the x-axis, respectively.

Additionally, an accurate center [$x_{center}$, $y_{center}$, $z_{center}]$ is a prerequisite for predicting an accurate bounding box. An accurate width along the y-axis and an accurate height along the z-axis also contribute to maximizing the fall detection accuracy. Based on the above observations, the box-based loss function is expressed as

(10) $$\begin{array}{c} {Loss}_{box}={Loss}_{{IoU}_{3D}}+{Loss}_{{center}_{dis}}+{Loss}_{line}\\ \;\;=1-{IoU}_{3D}+\frac{d^{2}\left(b^{r},b^{GT}\right)}{{\left(l^{e}\right)}^{2}+{\left(w^{e}\right)}^{2}+{\left(h^{e}\right)}^{2}}\\ \;\;\;\;+\frac{d^{2}\left(w^{r},w^{GT}\right)}{{\left(w^{e}\right)}^{2}}+\frac{d^{2}\left(h^{r},h^{GT}\right)}{{\left(h^{e}\right)}^{2}} \end{array}$$

where $b^{r}$ and $b^{GT}$ are the center positions of the predicted box and ground truth box, respectively; $l^{e}$, $w^{e}$, and $h^{e}$ are the length, width, and height of the enclosing box, respectively; $l^{r}$, $w^{r}$, and $h^{r}$ are the length, width, and height of the predicted box, respectively; and $l^{GT}$, $w^{GT}$, and $h^{GT}$ are the length, width, and height of the ground truth box, respectively.

In summary, the 3D point-box hybrid regression loss consisted of a 3D bounding box IoU loss and a pointwise loss, which can be described as

(11) $${Loss}_{HyLoss}={Loss}_{point}+{Loss}_{box}$$

where ${Loss}_{point}$ is the position loss of the points for optimizing the IoU of the human body and the ground reflection points.

### 3.3. Feature Extraction

**Figure 10:**
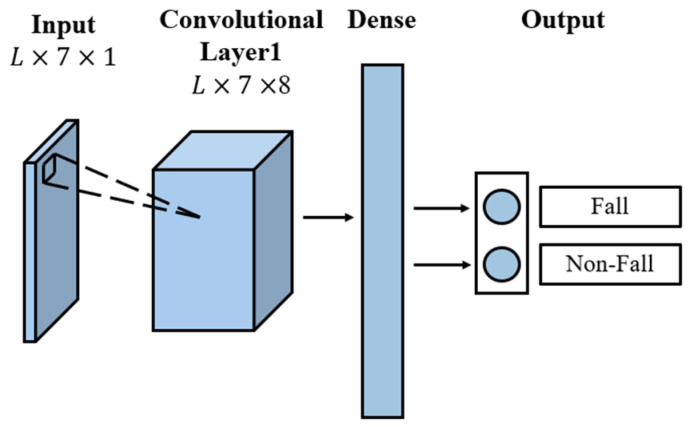
Architecture of the lightweight CNN.

We used a lightweight CNN for classification. The architecture of the lightweight CNN is shown in Figure 10. The CNN included an input layer sequence, one convolution layer, one dense layer, and a softmax layer. The feature parameters from one frame included $[x_{center}$, $y_{center}$, $z_{center},w,l,h,\theta ]$, and L frames of 7×1 feature parameters were first subjected to the convolution layer. The convolution layer captured the movement features, consisting of eight hidden neurons with a kernel size of eight. The convolution layer employed rectified linear units as activation functions for the hidden layers. The L×7×8 output from the convolution layer was fed into the dense layer. The softmax function was used in the final dense layer for classification.

## 4. Implementation and Evaluation

### 4.1. Setup and Data Collection

We used the 24 GHz mmWave FMCW radar from ICLegend Micro for data collection. The radar sensor had two transmitting antennas and four receiving antenna channels. The radar parameter configurations are listed in Table 1.

**Figure 11:**
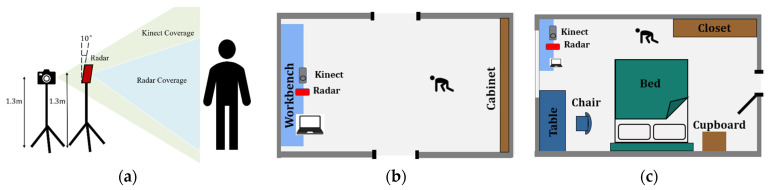
(**a**) Collected movement in dataset; (**b**) evaluation scenario: room A (office); (**c**) evaluation scenario: room B (bedroom).

To evaluate the proposed system, we set up the experiments and collected data in two different indoor environments. The experimental setup is shown in Figure 11. Room A, shown in Figure 11b, was a relatively empty office, and room B, shown in Figure 11c, was a bedroom where obstacles caused severe multipath and motion interference. The elevation field of view (FOV) of the Kinect sensor is 135 degrees, and the elevation FOV of the radar is 90 degrees. To capture more of a scene, the radar and Kinect sensors were both mounted at heights of 1.3 m. We collected 17,600 frames of data from 13 subjects performing fall and non-fall actions. The participants were aged between 20 and 30 years, and their heights were between 165 and 177 cm.

Furthermore, we divided these frames into three datasets:

Dataset0. This dataset included 10,860 frames from five participants who performed the experiments in room A. After data argumentation, including shifting, rotating, and adding noise points, the dataset included 13,560 frames.

Dataset1. This dataset included 3040 frames from four participants who performed the experiments in room A.

Dataset2. This dataset included 3700 frames from four participants who performed the experiments in room B.

For each dataset, the motion included falling backward and falling forward, sitting on a chair, jumping, walking, or squatting. The ratio of the number of fall samples to non-fall samples was 1:2.

### 4.2. Evaluation

In this section, we present the experimental results of the proposed model in a fall detection system. All algorithms were realized based on Python code. The computation platform used was a laptop with a 2.60 GHz Intel(R) Core (TM) i7-10750H CPU.

#### 4.2.1. Point Cloud Quality

**Figure 12:**
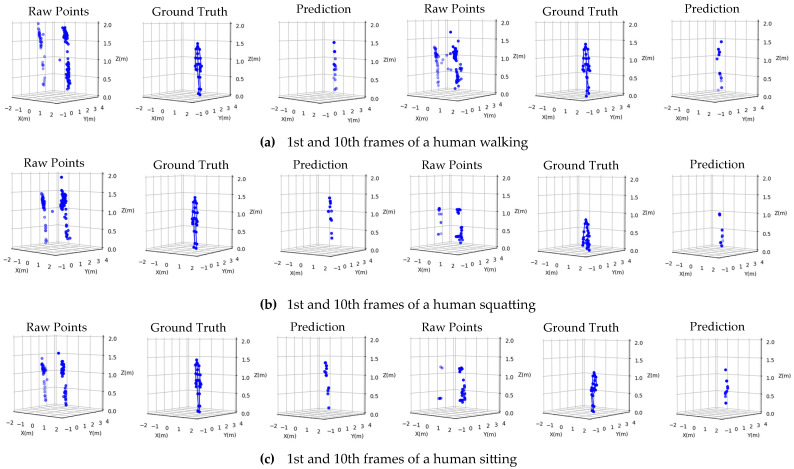

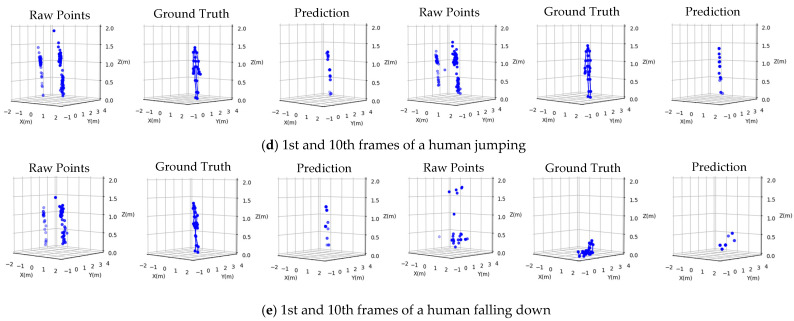
Comparison between raw point clouds, the ground truth, and the PCE model’s predicted points in two frames (1st and 10th) for five different actions (walking, squatting, sitting, jumping, and falling).

Before training the proposed PCE model, the 3D radar data and ground truth skeletal points from the Azure Kinect were extracted, as described in Section IV. Then, L frames of data from the radar were combined sequentially to obtain a 3D tensor (L×M×3). We then evaluated the PCE model for $L=\left\{1,2,\ldots 10\right\}$ by generating 10 distinct datasets, ${Dataset0}_{i}$, ${Dataset1}_{i}$ and ${Dataset2}_{i}$, where i $\in \left\{1,2,\ldots 10\right\}$ is the frame index. The proposed PCE model $M_{i}$ was trained on ${Dataset0}_{i}$, and the trained PCE model $M_{i}$ was subjected to ${Dataset1}_{i}$ and ${Dataset2}_{i}$, which did not participate in the training. Figure 12 shows predicted points. We selected two frames of each motion for comparison against the ground truth. In addition, because of the nature of the FMCW radar measurement, an exact and complete match of [*x*, *y*, *z*] between the predicted and ground truth keypoints was simply unrealistic; therefore, finding a method for evaluating the predicted results was crucial. 

To evaluate the quality of the point clouds, we calculated the average 3D IoU for every L frame scenario across the f test samples in the dataset according to the formula ${3DIoU}_{i}=\frac{1}{N}\sum_{j=1}^{N}{3DIoU}_{ij}$, $\forall i\in \left\{1,2,\ldots 10\right\}$. The average 3D IoU results for all L frames from Dataset1 and Dataset2 are outlined in Table 2 and Table 3, respectively. In the 10th frame, the average 3D IoU is lower than that in the other frames. The reason for this result was that sometimes the number of points was too small because the motion may have already been completed in advance. We also compared the raw point clouds from the radar and the DBSCAN method, which is the most popular method for removing outliers and improving the quality of point clouds. The proposed method outperformed the other methods. In addition, the PCE with the proposed HyLoss function outperformed the traditional MSE loss function.

**Figure 13:**
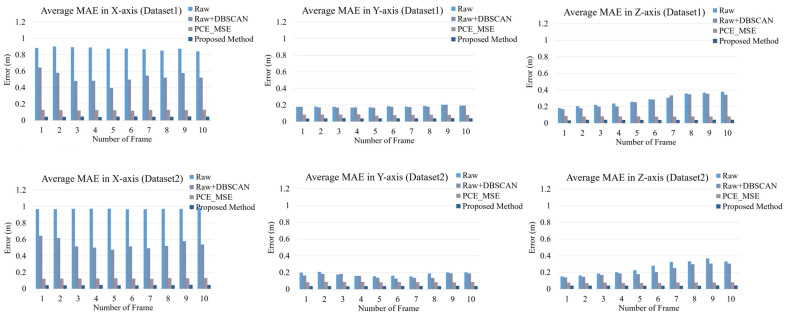
Localization error of the centroid of the points cloud compared to the baseline.

**Figure 14:**
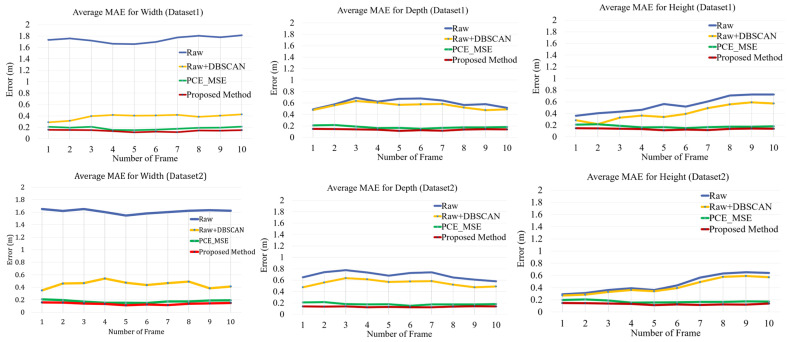
MAE of the width, depth, and height of the bounding box from the points cloud compared to the baseline.

Furthermore, we computed the centroids of the coordinates of the point clouds. For accuracy, the centroids of the coordinates of the predicted point clouds were compared with the centroids of the coordinates of the ground truth labels by calculating the mean absolute error (MAE) in the x-, y-, and z-coordinates. The centroid of the coordinates was $\left(\frac{max\left(x\right)+\min(x)}{2},\frac{max\left(y\right)+\min(y)}{2},\frac{max\left(z\right)+\min(z)}{2}\right)$. The average MAEs for all L frames tested in Dataset1 and Dataset2 are shown in Figure 13. We also compared the raw point clouds from the radar, DBSCAN, and PCE models using the traditional MSE loss function instead of the proposed HyLoss. The average MAE of the predicted method was comparable to that of the other methods. In other words, the localization of the centroid from the PCE model was the closest to the ground truth. In addition, we computed the average MAE of the width, depth, and height of the bounding box along the x-, y-, and z-axis. As shown in Figure 14, the width, depth, and height of the predicted bounding box were significantly lower than those of the other methods for both Dataset1 and Dataset2. This result indicates that the PCE model with the proposed HyLoss offers a better image of the human body for a wide range of people and environments. 

#### 4.2.2. Classification for Fall Detection

In terms of fall detection, the predicted high-quality point clouds of Dataset0, Dataset1, and Dataset2 were fed into the feature extractor to obtain the human pose parameters. The lightweight CNN shown in Figure 9 was used to classify normal and fall events. Dataset0 was used to train the lightweight CNN classifier, Dataset1 was used for validation with new people, and Dataset2 was used for validation with new people and new environments. Neither Dataset1 nor Dataset2 were involved in the entire training process. To validate the choice of PCE architecture parameters, we compared the average actuary, recall, and F1 of the PCE model with various parameters. The results are summarized in Table 4. Every encoder is denoted by E, and every decoder is denoted by D. For example, the proposed PCE model can be described as raw+32E+32E+16E+32D. Clearly, the proposed architecture is suitable. In addition, a comparison of the PCE with the traditional MSE loss function revealed that the accuracy, recall, and F1 score of the proposed HyLoss function outperformed those of the traditional function, which shows that the proposed HyLoss function improves the fall detection system.

The purpose of PCE is to improve the reliability of the classification during fall detection tasks. Therefore, we compared the performance of the PCE classification reliability (instead of position errors or IoU) with existing point cloud models. First, the predicted points are shown in Figure 9. The results are listed in Table 5. The accuracy of the raw points and DBSCAN method for Dataset0 were higher than 0.98 because the test data included some people in the training set. The accuracy of the raw points (baseline) and DBSCAN method for Dataset1 and Dataset2 were lower than 0.8 because the raw data from the radar detection contained many invalid points from new test data, and DBSCAN could not adequately remove a sufficient number of outlier points. However, the performance of the proposed method exhibited no obvious changes for any of the three datasets. This means that the point clouds predicted by the PCE could improve the performance of classification when new people and environments are involved. For computing the complexity analysis, the proposed method had 0.017 million parameters, which was a much smaller number than that of the other methods. The floating-point operations (FLOPs) associated with the proposed method was 0.303M, which was also lower than that of the other methods. In addition, the fall detection system achieved high accuracy and required less response time. For the response times of all the competing methods, we ran them on the same platform. Because all of them required the same computing time for feature extraction and classification, we only calculated the computing time of PCE on a single sample at a time. Although the accuracy was not favorable for new people and environments, the response time of the proposed method was shorter than that of the others. In summary, our work balances accuracy and speed.

However, fall detection based on radar currently uses different data formats, such as time-frequency maps and range-Doppler maps, and there is no public fall detection dataset for radar. It is difficult to find a baseline for a fall detection system; therefore, we compared the reported performance with that of other studies. As shown in Table 6, the proposed system achieves better performance for new people and environments. Although the performances of studies [22,40] was above 0.9, they were tested only on the same people and in the same environment. The performance of these studies may vary for new people and environments in a way similar to the results of the DBSCAN method shown in Table 5. In addition, although CW radar has lower cost, the accuracy of the proposed FMCW fall detection system was 0.989, which was higher than the CW radar system [41,42]. Moreover, even though the 77 GHz 3T4R radar has a higher resolution than our sensor, the proposed fall detection system could still outperform it [22].

To investigate the potential of our fall detection system for real-time implementation, we evaluated the computational time of one sample for all the steps in our work. As shown in Table 7, although the computing time for the PCE and classification was 20.349 ms in total, the signal processing cost a significant amount of time because, in this study, we used the super-resolution algorithm MUSIC for DOA, which is considered to be the bottleneck of real-time fall detection systems. However, further research is required to design a system with lower computing costs for real-time detection and mobile devices. Furthermore, it is difficult to collect samples when real falls start among older adults. The limitations of the sample size may cause potential biases. In the future, we will improve our experiment and test it with large-scale experiments in more practical environments and with more subjects.

## 5. Conclusions

This study demonstrated a fall detection system based on the 3D point clouds of a 24 GHz FMCW radar. Unlike other conventional methods that utilize more costly hardware to improve detection accuracy, we used a low-cost small radar antenna array operating at 24 GHz to maintain high accuracy. By applying the PCE model to a fall detection system, we improved the quality of the point clouds and also the system performance, especially the accuracy, sensitivity, and generalization ability for new users and environments. As a result of our efforts to reduce computing costs in signal processing, the proposed method has the potential for widespread applications in monitoring the health of the elderly in an indoor environment without considering privacy protection.

## Figures and Tables

**Table 1:** Unit parameter configuration of the radar.

Parameter	Value
Carrier frequency	24 GHz
Bandwidth	3.17 GHz
Duration of a chirp	460 μs
Number of chirps per frame	254
Chirps per CPI	64
Duration of a frame	117 ms

**Table 2:** Average 3D IoU of Dataset1 in each frame.

	1	2	3	4	5	6	7	8	9	10	Mean
Raw (baseline)	0.276	0.265	0.262	0.249	0.269	0.233	0.215	0.194	0.202	0.183	**0.234**
Raw + DBSCAN [26]	0.300	0.287	0.266	0.257	0.249	0.225	0.212	0.207	0.191	0.189	**0.259**
PCE_MSE	0.609	0.633	0.623	0.636	0.618	0.597	0.596	0.579	0.574	0.525	**0.599**
Proposed PCE_HyLoss	0.635	0.669	0.687	0.681	0.664	0.652	0.653	0.627	0.628	0.619	**0.651**

**Table 3:** Average 3D IoU of Dataset2 in each frame.

	1	2	3	4	5	6	7	8	9	10	Mean
Raw	0.300	0.287	0.266	0.257	0.249	0.225	0.212	0.207	0.191	0.189	**0.238**
Raw + DBSCAN [26]	0.276	0.265	0.262	0.249	0.269	0.233	0.235	0.224	0.222	0.193	**0.242**
PCE_MSE	0.591	0.613	0.625	0.614	0.612	0.628	0.591	0.571	0.574	0.526	**0.595**
Proposed PCE_HyLoss	0.649	0.673	0.677	0.697	0.652	0.665	0.624	0.629	0.614	0.618	**0.650**

**Table 4:** Classification result of the proposed PCE model using different architectures.

	Dataset0			Dataset1			Dataset2		
	Acc	Recall	F1	Acc	Recall	F1	Acc	Recall	F1
64E + 64E + 32E + 32D	0.993	0.994	0.995	0.976	0.977	0.977	0.972	0.973	0.972
16E + 16E + 16E + 32D	0.986	0.984	0.985	0.971	0.973	0.973	0.971	0.973	0.972
32E + 32E + 16E + 16E + 32D	1.000	0.994	0.994	0.991	0.993	0.993	0.988	0.988	0.988
32E + 32E + 16E + 32D + 32D	1.000	0.990	0.989	0.989	0.989	0.989	0.986	0.987	0.988
PCE_MSE (with traditional loss)	0.997	0.995	0.995	0.975	0.976	0.976	0.970	0.971	0.972
PCE with HyLoss (ours)	0.993	0.992	0.993	0.991	0.992	0.993	0.989	0.988	0.988

**Table 5:** Classification performance of same datasets using current point cloud generation methods.

	Params	FLOPs	Response	Dataset0			Dataset1			Dataset2		
	(M)	(M)	Time (ms)	Acc	Recall	F1	Acc	Recall	F1	Acc	Recall	F1
Raw	--	--	--	0.992	0.993	0.992	0.783	0.766	0.772	0.798	0.783	0.789
Raw + DBSCAN [26]	--	--	--	0.991	0.990	0.991	0.798	0.811	0.805	0.781	0.800	0.794
PointNet [27]	0.815	119	25.331	0.972	0.971	0.971	0.964	0.967	0.967	0.954	0.954	0.955
PCN [28]	4.430	4339	160.390	1.000	1.000	1.000	0.987	0.988	0.989	0.981	0.982	0.983
TopNet [37]	6.193	1916	149.867	0.995	0.996	0.995	0.988	0.987	0.987	0.986	0.985	0.984
GRNet [38]	64.938	10,962	4266.643	1.000	1.000	1.000	0.971	0.972	0.973	0.978	0.978	0.978
RFNet [39]	2.369	6532	434.403	0.996	0.995	0.995	0.991	0.991	0.990	0.989	0.989	0.988
PCE (ours)	0.017	0.303	20.326	0.993	0.992	0.993	0.991	0.992	0.993	0.989	0.988	0.988

**Table 6:** Comparison between the proposed method and other fall detection methods that use radar.

Study	Sensor	Data Format	Test Dataset		Performance
			New Environment	New People	
[22]	FMCW 77 GHz 3T4R	Point clouds	No	No	Accuracy: 0.98
[40]	FMCW 24 GHz 2T4R	Time-frequency map	No	No	Recall: 0.95 F2: 0.92
[41]	CW 25GHz	Time-frequency map	No	No	Accuracy: 0.825
[42]	CW 24GHz	Root mean-squared of signal	No	No	Accuracy: 0.977 Recall: 0.90
ours	FMCW 24 GHz 2T4R	Point Clouds	Yes	Yes	Accuracy: 0.989 Recall: 0.988 F1: 0.988

**Table 7:** Response time for each step in the system.

Step	Computing Time
Signal processing	6204.3 ms
CPE model	20.32 ms
Classification	0.029 ms

## Data Availability

Data are contained within the article.
